# The Relationship Between the Actual Level of Air Pollution and Residents’ Concern about Air Pollution: Evidence from Shanghai, China

**DOI:** 10.3390/ijerph16234784

**Published:** 2019-11-28

**Authors:** Daxin Dong, Xiaowei Xu, Wen Xu, Junye Xie

**Affiliations:** School of Business Administration, Southwestern University of Finance and Economics, Chengdu 611130, China; dongdaxinedu@126.com (D.D.); xxww@smail.swufe.edu.cn (W.X.); johnny@smail.swufe.edu.cn (J.X.)

**Keywords:** air pollution, public concern, air quality index, Baidu index, Shanghai

## Abstract

This study explored the relationship between the actual level of air pollution and residents’ concern about air pollution. The actual air pollution level was measured by the air quality index (AQI) reported by environmental monitoring stations, while residents’ concern about air pollution was reflected by the Baidu index using the Internet search engine keywords “Shanghai air quality”. On the basis of the daily data of 2068 days for the city of Shanghai in China over the period between 2 December 2013 and 31 July 2019, a vector autoregression (VAR) model was built for empirical analysis. Estimation results provided three interesting findings. (1) Local residents perceived the deprivation of air quality and expressed their concern on air pollution quickly, within the day on which the air quality index rose. (2) A decline in air quality in another major city, such as Beijing, also raised the concern of Shanghai residents about local air quality. (3) A rise in Shanghai residents’ concern had a beneficial impact on air quality improvement. This study implied that people really cared much about local air quality, and it was beneficial to inform more residents about the situation of local air quality and the risks associated with air pollution.

## 1. Introduction

According to the World Health Organization (WHO), 91% of the world’s population lives in areas where air pollution exceeds the safety limits [1]. Air pollution negatively affects both human’s daily life, such as emotional and physical health, and sustainable economic growth, such as labor productivity and tourism (e.g., [2,3,4,5]). However, air pollutants are not always visible, which might lead to the public being unaware of pollution. Prior studies have indicated that the public perception of air pollution might be inconsistent with objective air quality, which was evaluated using scientific indices such as PM${}_{2.5}$ and PM${}_{10}$ [6]. A potential reason is that individuals’ perceived air quality could differ on the basis of their sociodemographic status, including gender, age, education, knowledge, and health status (e.g., [7,8]). Therefore, perhaps there exists a gap between objective and subjective measures of air quality.

There are two important reasons why the relationship between the actual level of air pollution and residents’ concern about air pollution should be examined. On the one hand, ignorance or underestimation of the severity of air pollution potentially poses a threat to residents’ health since it could increase the probability of long term exposure to air pollutants. For instance, it was reported that 90% of residents in Hong Kong would not stop their outdoor activities in the face of poor air quality [9]. Whether residents’ concern about air pollution could correctly reflect the actual pollution level has become a critical question. On the other hand, a high level of public awareness regarding air pollution might contribute to political and social enthusiasm for the enforcement of environmental protection behaviors and policies. Larson [10] reported that the concurrent rise of online platforms in China has become a positive force for environmental data transparency in China. Kay et al. [11] provided further evidence showing that social media are capable of empowering the government to respond to the air pollution problem to ensure social stability. More recently, Lu et al. [12] stressed that public concern about air quality might have a more direct impact than perceived air pollution in influencing people’s behaviors and the actions of the community, production sectors, and the government. Therefore, how Chinese residents responded to air pollution should be carefully examined. In particular, more studies are needed to analyze the relationship between the actual level of air pollution and residents’ concern about air pollution, given the importance of public concern in shaping public health and environmental regulation practices.

The purpose of this study is to examine whether and to what extent actual air pollution is correlated with residents’ concern about air quality in Shanghai, China. Shanghai was chosen as the target city in this study since it is one of the largest and most developed cities in China. Although Shanghai has a high level of economic and social development, its air pollution issue is considerable. According to the Shanghai Environmental Bulletin published by the local government, during the year 2018, air quality was classified as “good” on only 93 days. In 2017 and 2016, the numbers were 58 and 78, respectively. By the end of 2018, Shanghai’s air pollution was even reported to be worse than that of Beijing [13], which is well known for its air pollution problem. However, it seems that most attention has been centered on air pollution issues in Beijing in recent years. Public opinion on air pollution from Shanghai residents should be examined since it might maintain pressure on governments to roll out environmental regulations to reduce air pollution effectively and efficiently. From another perspective, understanding Shanghai residents’ concern about pollution is valuable in providing residents with useful advice about public health and environmental protection.

In this study, actual air quality was measured by the air quality index (AQI) reported by environmental monitoring stations. Residents’ concern about air pollution was measured using the Baidu index for the online search keywords “Shanghai air quality”. Baidu index data were provided by Baidu, which is the most popular Internet search engine in China. The index was calculated on the basis of the search volume for a specific search item on a daily basis within a specific region. The Baidu index has been widely used to predict public health issues (e.g., [14,15,16,17]) and tourism flows (e.g., [18,19,20]) in China. Similar to its usage in public health and tourism studies, the Baidu index is also applicable in measuring the degree of public concern about air pollution [12]. This point will be discussed in detail later in the literature review section.

**Figure 1:**
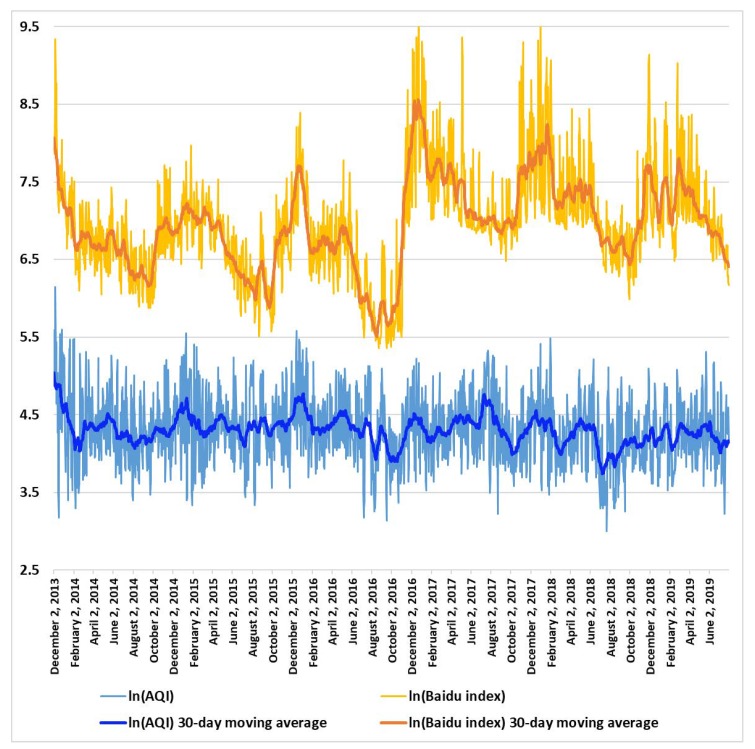
Actual air pollution and residents’ concern about air pollution in Shanghai from 2 December 2013 to 31 July 2019.

Figure 1 shows the logarithmic values of the actual AQI (blue curve) in Shanghai and the Baidu index (yellow curve) for “Shanghai air quality”. The sample period spanned from 2 December 2013 to 31 July 2019, covering 2068 days. The 30-day moving average values of the variables are also displayed to more clearly show varying trends. It can be observed that both the AQI and the Baidu index followed a similar cyclical pattern with apparent fluctuations. The correlation coefficient between logarithmic AQI and the Baidu index was 0.432, indicating a statistically significant positive correlation.

Although Figure 1 provides preliminary visual evidence on the relationship between actual air pollution and residents’ concern about air pollution, a basic correlation analysis is not sufficient. To better understand the relationship, the following three research questions are proposed. (1) To what extent and how soon could the actual air pollution level influence public concern about air quality? (2) Does a decline in air quality in another major city, such as Beijing, affect public concern about air quality in Shanghai? (3) Could public concern about air quality and the level of actual air pollution reciprocally influence each other? A vector autoregression (VAR) model was applied to answer these research questions due to its high ability to capture linear interdependencies among multiple variables over time.

It is expected that this study could add to the air pollution literature by examining the reciprocal interactions between public concern about air pollution and the actual degree of air pollution. From a practical perspective, it could help the municipal government of Shanghai better understand the degree of public concern about air quality and better assess the current environmental management practices. This study also provides insights into the spillover effects that the actual air pollution in other major tourist cities in China might have on public concern about air quality in Shanghai.

The rest of this paper is organized as follows. Section 2 presents a literature review and develops the hypotheses. Section 3 describes the empirical model and the data used in the analyses. The estimated results of the empirical model are reported in Section 4. Section 5 discusses the main findings and implications of the results. Section 6 concludes the paper and talks about limitations and directions for future research.

## 2. Literature Review and Hypothesis Development

### 2.1. Air Pollution: Actual Level, Perceived Level, and Public Concern

Actual air quality levels are measured by scientific techniques, and they are reported in the forms of different air pollutant indicators such as PM${}_{2.5}$ (particulate matter with a size of 2.5 micrometers or less) and SO${}_{2}$ (sulfur dioxide), or comprehensive indices such as AQI and API (air pollution index) constructed using the air pollutant indicators. Generally speaking, there is not much controversy on the proper measurement of actual air quality level, and scientists can measure it objectively and accurately.

People’s perception of air quality is subjective. Traditionally, researchers have used questionnaire surveys to collect data on perceived air quality level and people’s displeasure with air pollution (e.g., [21,22,23]). Some of the literature reported that perceived and actual levels of air quality are strongly correlated. For example, Atari et al. [24] conducted a community health survey in the “Chemical Valley” in Sarnia, Ontario, Canada, and they observed a significant correlation between odor annoyance scores and modeled ambient pollution. Peng et al. [22] analyzed the data for over 5000 valid respondents in 62 cities, on the basis of the China Social Survey 2013, and reported a congruence of perceived and actual air pollution. Similar findings were reported by some other studies, such as for Northeast England [25], for Switzerland [26], and for China [27].

However, some of the literature found that perceived air quality is not strongly related to actual air quality. For instance, Egondi et al. [21] focused on two slums in Nairobi, Kenya, on the basis of a cross-sectional study of 5317 individuals. They reported that the majority of respondents were exposed to air pollution. However, the perceived air pollution level was low among residents. This implied the need for promoting public awareness on air pollution. Focusing on the city of Wuhan in China, Guo et al. [7] reported that most persons they surveyed believed that air quality had become worse, though statistics of measured air pollutants over the period of 2010–2014 did not actually show that trend. During the period of 2005–2006, Semenza et al. [23] surveyed the residents of two U.S. cities, Portland and Houston. They reported that the residents’ perception of poor air quality was not related to PM${}_{2.5}$ or ozone indicators. On the basis of a sample of 200 people interviewed in London in 1999, Williams and Bird [28] reported that public perception of air quality was not a reliable indicator of actual air pollution levels in the investigated areas. The inconsistency between actual and perceived level of air quality was also reported by Berezansky et al. [29] for Haifa, Israel, by Brody et al. [30] for Texas in the U.S., and by Kim et al. [31] for Seoul, South Korea.

A notable recent study was by Lu et al. [12]. They used the correlation-analysis technique to study the relationship between actual PM${}_{2.5}$ concentration and public concern about haze in five large Chinese cities for the period between 2013 and 2017, where public concern was measured by the Baidu index. They reported that short term fluctuations in actual pollution level and concern about pollution were strongly and positively correlated, but long term annual trends of these two variables were opposite.

Overall, the literature has not provided a consensus on whether the public could correctly perceive air quality and whether public concern about air pollution concurs with the actual level of air pollution. There are two possible explanations of this disagreement in the literature. First, different studies were based on different samples. Since social and individual characteristics strongly affect people’s viewpoints [31,32,33], it is natural to see that perceptions of some groups are consistent with actual air quality, but some other groups tend to over- or under-estimate the level of air pollution. Second, public perceptions vary over time. Along with the development of information technology, improvements in education, and the propagation of mass media, the public receives increasing levels of information on the current situation and importance of air quality. Therefore, many people might not have perceived and been concerned with air pollution problems in the past, but they have changed their minds in recent years.

In this study, we focus on the city of Shanghai. Shanghai is one of the most important and developed cities in China. In 2018, Shanghai’s GDP per capita exceeded 20,000 U.S. dollars, which was close to that of Greece and Slovakia. Shanghai has a large population size of over 24 million residents, close to the population of a middle sized country such as Australia. As the financial center of China, many companies have their headquarters in Shanghai. Shanghai has good educational resources, including 64 universities. As an international city, Shanghai is visited by approximately nine million (person-times) inbound tourists annually. Therefore, on average, Shanghai residents have the knowledge and ability to perceive the importance of clean air and to worry about pollution if they feel that air quality has declined. Accordingly, the first research hypothesis in this study was established:

**Hypothesis** **1.** Deprivation of air quality in a local area raises the public concern about the air pollution problem.

Furthermore, since the Internet has allowed information to flow swiftly across regions, news about air pollution in one major city could quickly propagate and might cause mental or even physical responses of residents in another city. In this study, Beijing, the capital of China, was used as a study area because it is notorious for its severe air pollution (it is also important to note that other major tourist cities, including Nanjing and Guangzhou, are also examined in the robustness check section). If Beijing’s AQI rose to an unhealthily high value, the residents in Shanghai might also want to check the air quality in their local area after they receive the information about Beijing. Thus, the second research hypothesis was built:

**Hypothesis** **2.** Deprivation of air quality in another major city raises public concern about the air pollution problem in the local city.

### 2.2. Importance of Air Pollution Awareness and Concern

People’s concern about air pollution is largely due to the health risks it has [21,34,35]. People’s concern about air quality promotes people’s avoidance of the polluted environment. For instance, people tend to limit their social activities on polluted days [36]; people are unwilling to buy houses located in polluted areas [29,37,38]; and tourists flow from polluted to less polluted cities [39,40].

Public concern about air pollution generates pressure on the government to implement policies to promote better air quality. Environmental perception and concern also promote more pro-environmental behaviors [41,42]. Tam and Chan [43] further examined the association between environmental concern and behavior across 32 countries (China was not included), and they indicated that the association was weaker in countries with stronger distrust and belief in external control. To better understand the strength of this association in the case of Shanghai, the third research hypothesis was built:

**Hypothesis** **3.** Public concern about the air pollution problem pushes people or governments to take actions to reduce air pollution.

### 2.3. Applicability of the Baidu Search Index to Measure Residents’ Concern about Air Pollution

Internet search engines enable quick access to information. Do et al. [44] indicated that tracking online search behavior using relative search volumes was an effective way to gauge public interest. A number of researchers have used the search index as a proxy of issue salience or attention in the public. The majority of search-volume data are extracted from two dominant engines, Google and Baidu. For example, Caputi et al. [45] noticed a significant public interest in heat-not-burn tobacco products on the basis of Google trends data. Do et al. [44] utilized Google trends data to assess public awareness on protected wetlands in South Korea. Mellon [46] found that Google trends data could be used to measure the salience of four issues (fuel prices, economy, immigration, and terrorism) in the United States. Search index data have also been used to predict tourism flows (e.g., [18,19,20,47]).

Given the fact that the Baidu search engine occupies a dominant market share in China, the Baidu search index has become a useful proxy for Chinese residents’ interest and concerns. In Li et al. [16], Baidu search query data were found to be a reliable indicator for monitoring and predicting the HIV/AIDS epidemic in China. Liu et al. [48] used the Baidu index to assess people’s awareness of avian influenza A(H7N9) in Zhejiang, China. Using the Baidu index, Yang et al. [49] found that the daily average PM${}_{2.5}$ concentration had a weak impact on public awareness of lung cancer risk in China. Lu et al. [12] used the Baidu index to measure public concern about haze in five large Chinese cities. Given that the use of the search index has been extensively applied in multiple disciplines as a method for surveillance, monitoring, and measuring interest on and concern about specific topics, this study adopted Baidu index data to measure residents’ concern about air pollution in China.

## 3. Methods

### 3.1. Model

This study is based on time series data relevant to air pollution in Shanghai. VAR analysis, a powerful tool in modeling complex time series, was used in this study. Beyond basic correlation analysis or the ordinary least squares (OLS) regression technique, the VAR model treats all variables as endogenous and contains time lagged variables. Thus, the VAR model can measure the reciprocal reactions among different variables and lagged and persistent effects. The following reduced form VAR model was built:

(1) $$y_{t}=c+\underset{i=1}{\sum^{p}}A_{i}y_{t-i}+\varepsilon_{t},$$

where $y_{t}$ is a vector of endogenous variables in period *t*, c is the intercept vector, $A_{i}$ refers to the autoregressive coefficient matrix that captures system dynamics, and $\varepsilon_{t}$ is the residual term. Lag order *p* of the model is selected on the basis of some statistical criteria that are discussed later.

Besides the variables of AQI and the Baidu index in Shanghai, the AQI in Beijing was also included in the model as a potential explanatory variable that might affect Shanghai residents’ concern about air pollution. Indeed, in modern times, information is highly mobile via different channels. People’s views and opinions are impacted by not only situations in their local area but also by things occurring in other districts.

Therefore, in this study, vector $y_{t}={\left[AQI_{t},AQI_{t}^{Beijing},BaiduIndex_{t}\right]}^{{}^{\prime }}$ was used. It contains three variables: $AQI_{t}$, daily air quality index in Shanghai; $AQI_{t}^{Beijing}$, air quality index in Beijing; and $BaiduIndex_{t}$, the Baidu index for keywords “Shanghai air quality”. As usual, all three variables were log transformed to mitigate potential scaling problems. Hence, variable variations are expressed as percentage changes.

### 3.2. Data for Measuring Actual Air Pollution

Daily AQI data used to measure actual air pollution level in Shanghai are publicly available from the website of the China Air Quality Online Monitoring and Analysis Platform: https://www.aqistudy.cn. AQI is a synthesized index reflecting the degree of pollution in ambient air, calculated using the measured data for several major air pollutants (PM${}_{2.5}$, PM${}_{10}$, CO, NO${}_{2}$, O${}_{3}$, SO${}_{2}$) according to the guidelines of the official environmental protection sector. A low AQI value indicates a low degree of air pollution, and a high AQI value implies a high degree of air pollution.

The daily AQI values of 2068 days over the period between 2 December 2013 and 31 July 2019 in Shanghai were retrieved to measure actual air pollution in Shanghai. Additionally, in order to answer the second research question of whether the decline in air quality in another major city, such as Beijing, affects the public concern about air quality in Shanghai, the daily AQI data of Beijing during the same period were also retrieved. Data prior to 2 December 2013 were not included in this study due to data unavailability.

### 3.3. Data for Measuring Residents’ Concern about Air Pollution

The Baidu index was utilized to measure residents’ concern about air pollution in Shanghai. The Baidu index data were provided by Baidu, Inc., and they are publicly available from the web page: http://index.baidu.com. Baidu is the most popular Internet search engine that occupies a major market share in China. The Baidu index is calculated on the basis of the search volume for a specific search item on a daily basis at the municipal, provincial, and national levels [16]. A high (low) value of the Baidu index for a certain item indicates that many (few) persons searched for information on the item and cared about the relevant topic. Compared to self-administrated survey methods, the Baidu index has two advantages in measuring the degree of public concern about air pollution. First, it covers a wide range of study samples. Since Baidu dominates the search engine market in China, its Baidu index could reflect the aggregate behaviors of most Chinese Internet users. Second, the Baidu index data are available at a daily frequency for several years. This property enabled us to examine not only the long term trend, but also short term fluctuations of public concern about air pollution.

In this study, the Baidu index was restricted within the district of Shanghai in order to rule out individuals who were not living in Shanghai, but were still interested in Shanghai’s air quality. The keywords “Shanghai air quality” (“shanghai kongqi zhiliang” in Chinese) were selected as the searched-for item. Other related search terms, such as “Shanghai haze” (“shanghai wumai” in Chinese) and “Shanghai PM${}_{2.5}$” (“shanghai pm2.5” in Chinese) were also tested for robustness checks. Consistent with the sample period for the AQI data, the Baidu index data between 2 December 2013 and 31 July 2019 were exploited.

Table 1 shows the summary statistics of the variables used in the analyses. In the table, both the original level and logarithmic value of variables are shown. In the VAR estimation, the logarithmic values were used to mitigate potential scaling problems.

## 4. Results

This section reports the results of the empirical analyses. This section first explains how the optimal lag order for the VAR model was selected. Then, this section reports the core estimation results, focusing on the impulse response figures of different variables and forecast error variance decomposition (FEVD) estimates. After that, several robustness checks on the results are conducted.

### 4.1. Selection of Optimal Lag Order

Before estimating a VAR model, it is necessary to choose the optimal lag order for the model on the basis of lag order selection statistics. Table 2 reports useful statistics. It shows that different criteria suggest different selections of lag order. The LR (likelihood ratio), FPE (final prediction error), and AIC (Akaike’s information criterion) statistics suggested to use eight lags. Differently, the HQIC (Hannan and Quinn information criterion) suggested six lags, and SBIC (Schwarz’s Bayesian information criterion) suggested three lags. Since three of these five criteria suggested to use eight lags in the model, eight lags were initially selected for VAR estimation. In the robustness check section, situations using three and six lags are examined, and they demonstrated robust results.

### 4.2. Estimation Results

**Figure 2:**
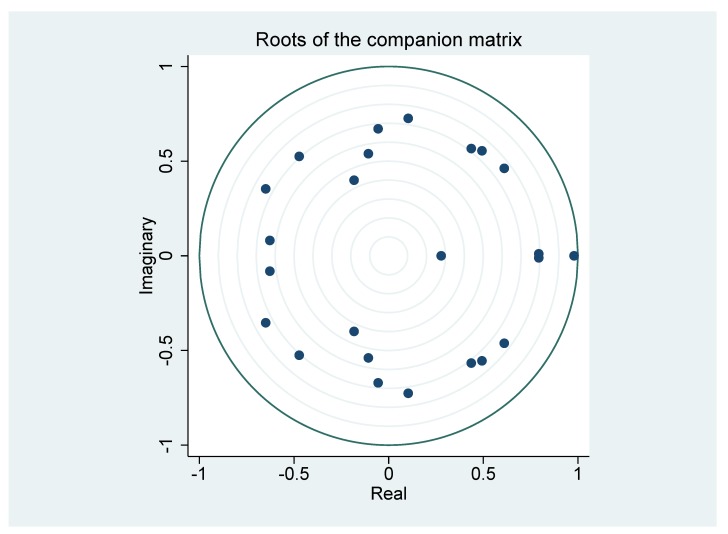
Eigenvalue stability condition.

In order to generate meaningful estimation results, the whole VAR system must be stable. Figure 2 shows that all eigenvalues were inside the unit circle. This indicates that the established VAR system satisfied the required stability condition, and it could be relied on to analyze interactions among variables.

**Figure 3:**
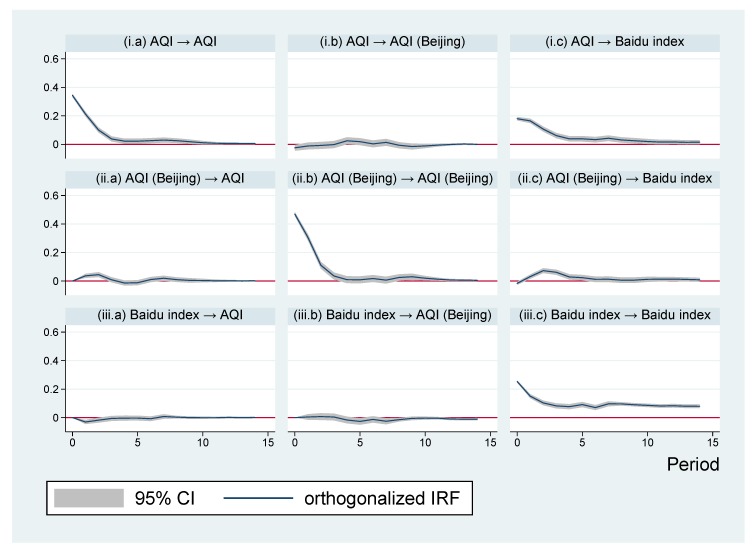
Impulse response figures (IRFs). Note: Each subfigure with the title of “X→Y” demonstrates the response of variable Y to an orthogonalized positive shock of variable X. In other words, X is an impulse variable, and Y is a response variable. One period in the figure denotes one day.

Since the estimation results of a VAR model are rarely only explained by the estimated coefficients, the analyses primarily focused on impulse response figures (IRFs). Figure 3 shows the impulse response figures based on the estimated coefficients.

**Figure 4:**
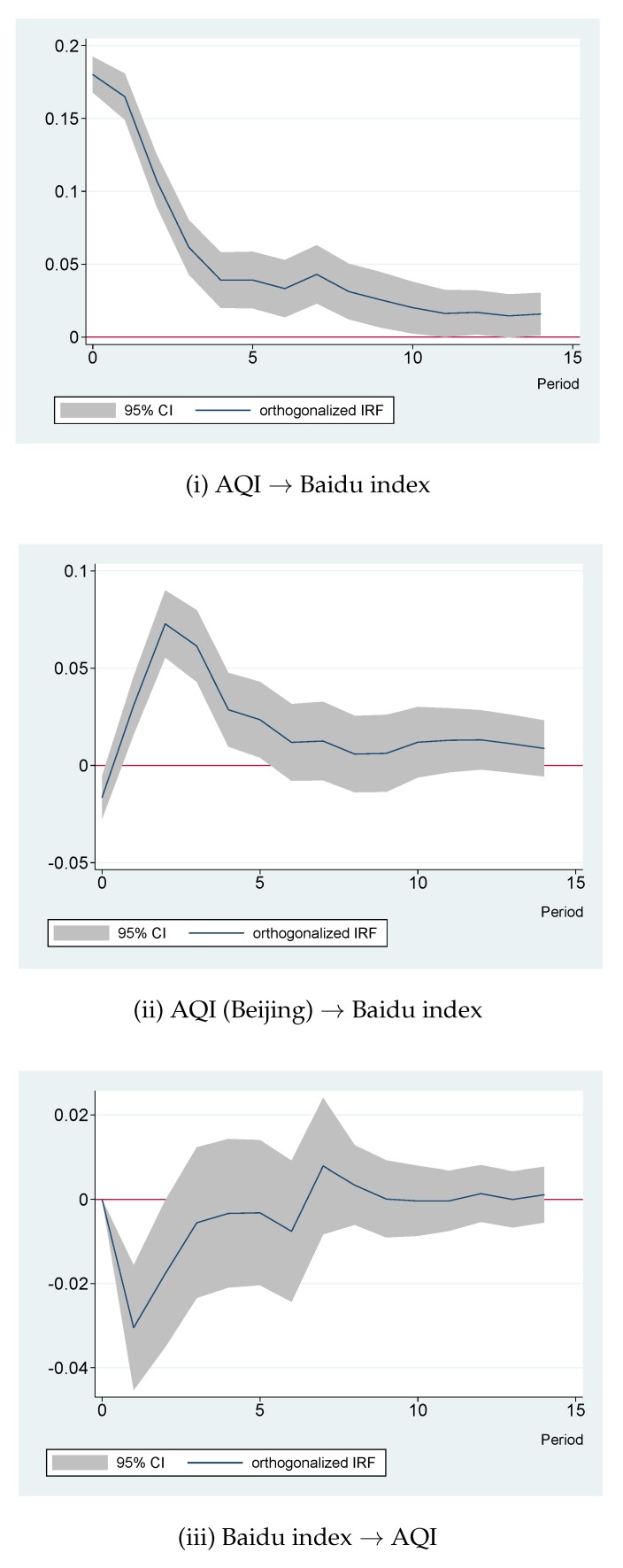
Amplified impulse response figures (IRFs) of interest. Note: Each subfigure with the title of “X→Y” demonstrates the response of variable Y to an orthogonalized positive shock of variable X. In other words, X is an impulse variable, and Y is a response variable. One period in the figure denotes one day.

The three subfigures in the first row of Figure 3 show the responses of variables to an orthogonalized, one unit, positive shock of (logarithmic) AQI in Shanghai. As shown in Figure 3(i.a), after such an AQI shock, AQI notably increased. As displayed in Figure 3(i.b), the AQI in Beijing fluctuated a little bit, but variation was quite small and not statistically significant. Figure 3(i.c) notably shows that, in response to the AQI shock, the Baidu index rose significantly. To observe the response more clearly, this subfigure was amplified and is displayed in Figure 4(i). It is clear that the AQI shock immediately raised the Baidu index without any time lag. The response was persistent and significantly positive even after ten days. Therefore, Hypothesis 1 is supported. The residents in Shanghai really cared much about the ambient air quality. As air quality deteriorated, Shanghai citizens expressed more concerns about local air pollution, as reflected by the increase in the Baidu index.

The three subfigures in the second row of Figure 3 show the variable responses to a positive shock of (logarithmic) AQI in Beijing. In Figure 3(ii.a), an increase in Shanghai’s AQI was observed. This could be explained by the spatial spillover effects of air pollution, as discussed in previous studies reporting the spatial interactions of air pollution among different regions (e.g., [50,51,52]). Figure 3(ii.b) shows that Beijing’s AQI increased persistently. An important finding was that, as demonstrated by Figure 3(ii.c), the Baidu index for Shanghai’s air quality rose in response to air pollution in Beijing. This implied that Shanghai residents’ concern about local air quality increased after they observed that Beijing’s air quality became worse. This graph was amplified and is displayed in Figure 4(ii). As demonstrated in that graph, one day after Beijing’s AQI increased, the Baidu index for Shanghai’s air quality began to increase. This increase was persistent for five days. The Baidu index returned to its original value after the sixth day. Therefore, Hypothesis 2 is supported. Air pollution problems in another large city indeed tend to increase the residents’ concern about air pollution in Shanghai.

The subfigures in the last row of Figure 3 demonstrated the variable responses to a positive shock of the (logarithmic) Baidu index. Figure 3(iii.a) presents the interesting finding that the AQI in Shanghai actually decreased after the Baidu index increased. In order to observe the details more clearly, this impulse response figure was amplified in Figure 4(iii). As can be seen from the graph, one day after an increase in the Baidu index, the AQI in Shanghai was below its initial level. This phenomenon lasted for two days. After that, the AQI returned back to its initial level. This finding supports Hypothesis 3. If public concern about air pollution intensified, people would take action to ameliorate air quality or, at least, avoid aggravating pollution and wait for the air quality to naturally improve. Figure 3(iii.b) demonstrates that the Baidu index had no significant impact on Beijing’s AQI. Figure 3(iii.c) demonstrates the persistency of the increase in the Baidu index.

Table 3 shows FEVD estimates for the Baidu index. As can be seen from the table, generally, within the horizon of fourteen days, local AQI explained around 30%–40% forecast error variance of Shanghai residents’ concern about air pollution. The AQI in Beijing explained roughly 5% variance. The rest was explained by the Baidu index itself. Obviously, local air quality was quite important in forecasting fluctuations of the Baidu index. Air quality in another famous city could also partly explain changes in the Baidu index for Shanghai’s air quality. These support previous findings that were obtained by observing impulse response figures.

### 4.3. Robustness Analyses

**Figure 5:**
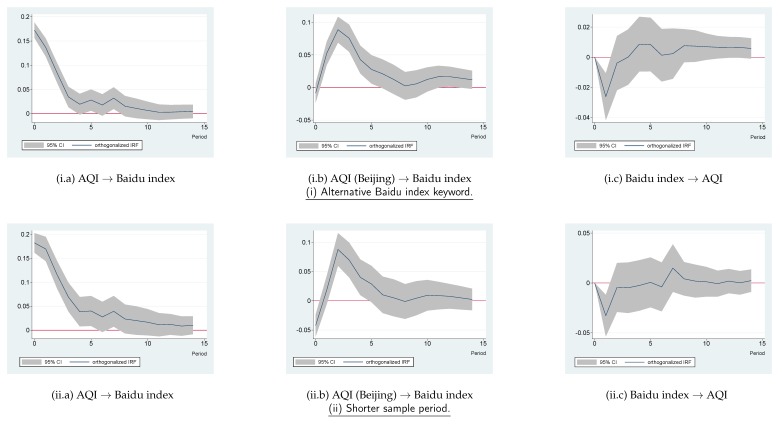

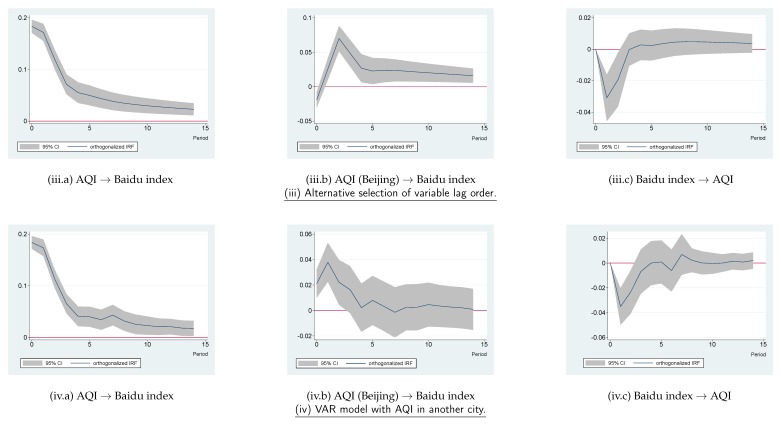
Robustness analyses: impulse response figures (IRFs). Note: Each subfigure with the title of “X→Y” demonstrates the response of variable Y to an orthogonalized positive shock of variable X. In other words, X is an impulse variable, and Y is a response variable. One period in the figure denotes one day.

In this subsection, we outline several robustness checks that were conducted on previous estimation results. First, whether results were sensitive to the selection of the search engine keyword for the Baidu index was examined. Second, alternative sample periods were considered. Third, alternative selections of lag orders in the VAR model were inspected. Lastly, whether air quality in other cities aside from Beijing affected the public concern about air pollution in Shanghai was further investigated. The impulse response figures are displayed in the subfigures of Figure 5.

#### 4.3.1. Alternative Baidu Index Keyword

In previous analyses, the keywords “Shanghai air quality” (“shanghai kongqi zhiliang” in Chinese) were relied on to derive the Baidu index. Next, another search term, “Shanghai haze (“shanghai wumai” in Chinese), was utilized to get the Baidu index. The results are shown in Figure 5(i.a–i.c). It is apparent that the AQI in Shanghai positively affected the Baidu index; the AQI in Beijing positively affected the Baidu index for Shanghai; and a rise in the Baidu index tended to depress the AQI in Shanghai. Thus, the previous findings of this study remained unchanged. In addition, other search terms such as “Shanghai PM${}_{2.5}$”, “air quality”, “haze”, and “PM${}_{2.5}$” were checked, and similar results were generated. To save space, the results using those alternative keywords are not reported here. The Supplementary Material attached to this paper provides additional information to demonstrate the robustness of the study results to the selection of Baidu index keywords. In the Supplementary Material, it is shown that the Baidu index values for different keywords were strongly correlated, indicating that different keywords actually reflected highly consistent online searching behaviors and provided similar information. Moreover, the Supplementary Material demonstrates the IRFs for the keywords “Shanghai PM${}_{2.5}$” (which had the lowest correlation coefficient with “Shanghai air quality”, compared to other candidate keywords), which were almost the same as the IRFs shown in Figure 3.

#### 4.3.2. Shorter Sample Period

The baseline analyses were based on the sample period between 2 December 2013 and 31 July 2019. It was admitted that the level of the Baidu index was not only determined by the degree of public interest on the specific topic, but also influenced by some other factors such as the changes in the market share of the Baidu search engine, total number of Internet users, and Internet users’ habits. The longer the sample period was, the larger the impact those alternative factors might have. As pointed out by Lu et al. [12], the long term annual trend of public concern about air pollution probably had characteristics different from those of short term fluctuations in air pollution. To mitigate this issue and inspect whether the study results were robust to the selection of the sample period, a shorter sample period from 1 January 2017 to 31 July 2019 was considered. Results are presented in Figure 5(ii.a–ii.c), which are similar to those that have previously been derived. Other subsample periods, such as between 1 January 2018 and 31 July 2019, were also checked. Results were analogous, but are not reported here to save space.

#### 4.3.3. Alternative Selection of Variable Lag Order

Previously, eight lags of variables were selected for the VAR model according to the LR, FPE, and AIC statistics. Since the HQIC and SBIC suggested to use different lags, the model was re-estimated on the basis of the alternative selections of lag order. According to the SBIC, three lags might be suitable. The corresponding impulse response figures are demonstrated in Figure 5(iii.a–iii.c). Notably, these new impulse response figures did not shake the previous statements in this study. Other lag orders, such as six, to follow the suggestion by HQIC, were also tested. Similar results were obtained.

#### 4.3.4. VAR Model with AQI in Another City

Previous analyses used a VAR model containing the AQI variable in Beijing. Next, whether results were sensitive to the selection of this specific city was checked. The city of Nanjing was taken instead of Beijing. Nanjing is one of the largest and most important cities in East China. The obtained impulse response figures are demonstrated in Figure 5(iv.a–iv.c). Compared to the baseline results, it was found that situations would be similar if Nanjing rather than Beijing were selected. Moreover, circumstances using the AQI in Guangzhou, which is the largest city in South China, were also inspected. Results were similar, but not reported here.

Overall, robustness checks strengthened the findings of this study. Hypotheses 1–3 were all supported.

## 5. Discussion and Implications

The estimation results in this study provided three interesting findings. First, local residents perceived the deprivation of air quality and expressed their concern about air pollution quickly, within the day on which the air quality index rose. This supported previous studies that found a strong correlation between perceived and actual level of air quality (e.g., [22,24]). It was implied that air quality is consistently monitored and assessed by Shanghai residents and that they show high awareness of air pollution. This also lends support to the finding by Yan et al. [36] that people who live in richer and more polluted cities are more likely to perceive air pollution. In addition, the concurrent rise of social media platforms in China might also contribute to this strong association. These platforms provide a way to share news and public opinions quickly. Media alerts on AQI would trigger heated debate and discussion on air quality, as well as information seeking behavior.

Second, a decline in air quality in another major city, such as Beijing, also raised the local concern about air quality in Shanghai. This was plausible because air pollutants could be transported by wind, causing pollution to spread over an extensive region within a short time interval. Prior studies have provided evidence that air pollution has a negative spatial spillover impact on neighboring cities’ public health [50]. Given the fact that Beijing is 1200 km away from Shanghai, air pollution in Beijing might not directly cause health problems in Shanghai. However, it increases public concerns on air pollution.

Third, a rise in Shanghai residents’ concern had a beneficial impact on air quality improvement. On the one hand, this could be explained by prior findings revealing that environmental concerns could promote people’s pro-environmental behaviors [41,42]. On the other hand, public concerns about air pollution could force governments to take actions to improve air quality [11,12]. It has been reported that China has curbed industrial emissions, restricted the use of cars on the road, and shut down coal mines in large cities such as Beijing, Shanghai, and Guangzhou [53].

This study contributed to the air pollution literature by empirically examining the reciprocal relationship between public concern about air quality and actual air quality using data on a daily basis. Different from prior studies that relied on survey data to measure perceived air quality, this study utilized a big data based dataset dating back to 2013 to conduct more accurate analyses. Additionally, this study performed VAR analysis rather than only basic correlation analysis, which helped demonstrate the rapidness and persistency of the rise in public concern about air pollution.

From a practical perspective, it was implied that providing timely air quality indices to residents could be a powerful tool to raise public awareness on air pollution to take steps for pollution mitigation. The government could utilize online search engines as a tool for displaying more information and advice explaining how people can minimize their contribution to air pollution during residents’ keyword search process. When observing a significant drop in air quality, the government should initiate immediate actions to tackle air pollution by providing information explaining how people can minimize their exposure to polluted air and which air pollution reduction strategies have been implemented in order to prevent excessive concerns on pollution. Furthermore, displaying AQI in other major Chinese cities could be utilized by local governments to promote local residents’ awareness of air pollution and, subsequently, support for environmental protection.

## 6. Conclusions, Limitations, and Future Research

Using Shanghai as a case study, this study empirically examined the interactive relationship between actual level of air pollution and residents’ concern about air pollution on the basis of the daily Baidu search index and AQI data. This study highlighted that residents in Shanghai expressed immediate concerns about air pollution as long as the air quality in Shanghai or in other major Chinese cities got worse. The study results also suggested that raising awareness on air pollution would motivate individuals or the government to carry out actions to improve air quality.

In evaluating the significant findings from this study, two major limitations need to be acknowledged. First, this paper focused on the circumstances in only one city because of difficulties in data collection. If we can collect data for a larger number of cities in the future and use the panel VAR approach, we could re-examine the findings based on Shanghai. A dataset covering more regions could also allow us to investigate possible heterogeneities across different regions, which may provide further policy implications. Second, as a preliminary exploration, this study only considered three variables in the VAR model because other variables were not available on a daily frequency. There is no doubt that some other factors may also be influential in the relationship between actual air pollution level and public concern about the problem. In the future, more variables may be introduced into the model as long as the data availability problem is overcome.

## Figures and Tables

**Table 1:** Summary statistics.

Variable		Observations	Mean		Std. Dev.	Minimum	Maximum
AQI	original	2068	81.27		38.95	20	468
	logarithmic	2068	4.30		0.44	3.00	6.15
$AQI^{Beijing}$	original	2068	108.24		68.01	21	500
	logarithmic	2068	4.51		0.58	3.04	6.21
BaiduIndex	original	2068	1351.33		1375.95	212	15,858
	logarithmic	2068	6.95		0.67	5.36	9.67

**Table 2:** Lag order selection statistics for vector autoregression (VAR) model. LR, likelihood ratio; FPE, final prediction error; AIC, Akaike’s information criterion; HQIC, Hannan and Quinn information criterion; SBIC, Schwarz’s Bayesian information criterion.

lag	LR	FPE	AIC	HQIC	SBIC
0		0.022541	4.72123	4.72424	4.72944
1	4890.7	0.002112	2.35356	2.36559	2.38638
2	177.43	0.001955	2.27609	2.29715	2.33353
3	72.068	0.001904	2.24981	2.27990	2.33188 *
4	62.367	0.001863	2.22826	2.26737	2.33494
5	43.078	0.001841	2.21607	2.26421	2.34737
6	42.971	0.001819	2.20394	2.26110 *	2.35986
7	34.426	0.001804	2.19596	2.26215	2.37649
8	28.796 *	0.001795 *	2.19071 *	2.26593	2.39587
9	11.875	0.001800	2.19369	2.27793	2.42346
10	13.575	0.001804	2.19584	2.28911	2.45023

Note: * indicates optimal lag order selection according to each statistic.

**Table 3:** Forecast error variance decomposition (FEVD) estimates for the Baidu index.

Forecast Horizon	FEVD of the Baidu Index		
	AQI	AQI (Beijing)	Baidu Index
0	0	0	0
1	0.334	0.003	0.663
2	0.404	0.008	0.588
3	0.406	0.037	0.557
4	0.395	0.054	0.551
5	0.386	0.056	0.558
6	0.374	0.056	0.570
7	0.369	0.055	0.576
8	0.358	0.053	0.589
9	0.347	0.051	0.603
10	0.337	0.049	0.614
11	0.328	0.048	0.624
12	0.320	0.047	0.633
13	0.312	0.047	0.641
14	0.305	0.046	0.649

## Supplementary Materials

The Supplementary Material is available online at https://www.mdpi.com/1660-4601/16/23/4784/s1.
