# Supplementary material for: The Relationship Between the Actual Level of Air Pollution and Residents’ Concern about Air Pollution: Evidence from Shanghai, China
Source: Int J Environ Res Public Health. 2019 Nov 28;16(23):4784. doi: 10.3390/ijerph16234784 (PMC6927008; doi:10.3390/ijerph16234784)
Supplement: Supplementary file 1 [file ijerph-16-04784-s001.pdf]

Supplementary Material to "The Relationship Between the Actual Level of Air Pollution and Residents' Concern about Air Pollution: Evidence from Shanghai, China"

Daxin Dong , Xiaowei Xu *, Wen Xu and Junye Xie

School of Business Administration, Southwestern University of Finance and Economics, Chengdu, China

* Correspondence: xiaoweix@swufe.edu.cn

The main text of the paper focused on the results by using the Baidu index of the search engine keyword "Shanghai air quality" to measure the degree of residents' concern about air pollution in Shanghai. This supplementary material provided additional information that the study results were not sensitive to the selection of Baidu index keywords.

Table S1 reported the Pearson correlation coefficients among the (logarithmic) Baidu index values of six different search engine keywords: "Shanghai air quality", "Shanghai haze", "Shanghai $PM_{2.5}$", "air quality", "haze", and "$PM_{2.5}$". From the table, it could be seen that except the correlation between "Shanghai air quality" and "Shanghai $PM_{2.5}$", all coefficients were much larger than 0.5, indicating a strong positive correlation. All coefficients in the table were statistically significant at the 1% level. Therefore, it was clear that different keywords essentially provided similar information on residents' concern about air pollution in Shanghai. There would be no surprise to see that the study results were robust to the selection of Baidu index keywords.

Table S1: Correlation coefficients among the (logarithmic) Baidu index values of different keywords.

	**Shanghai air quality**	**Shanghai haze**	**Shanghai $PM_{2.5}$**	**air quality**	**haze**	**$PM_{2.5}$**
**Shanghai air quality**	1					
**Shanghai haze**	0.612	1				
**Shanghai $PM_{2.5}$**	0.246	0.676	1			
**air quality**	0.740	0.841	0.700	1		
**haze**	0.640	0.849	0.581	0.805	1	
**$PM_{2.5}$**	0.680	0.871	0.682	0.949	0.869	1

Note: The Baidu index values were collected for different keywords in Chinese. The Chinese versions were "*shanghai kongqi zhiliang*", "*shanghai wumai*", "*shanghai pm2.5*", "*kongqi zhiliang*", "*wumai*", "*pm2.5*" for the terms "Shanghai air quality", "Shanghai haze", "Shanghai $PM_{2.5}$", "air quality", "haze", "$PM_{2.5}$", respectively.

To illustrate, Figure S1 demonstrated the impulse response figures (IRFs) for the VAR model with Baidu index of keyword "Shanghai $PM_{2.5}$" (which had the lowest correlation coefficient with "Shanghai air quality" as reported in Table S1). The IRFs were almost the same as the IRFs displayed in Figure 3 in the main text. Particularly, subfigure S1(i.c) showed that the Baidu index positively responded to the change in the AQI in Shanghai. Subfigure S1(ii.c) showed that the Baidu index in Shanghai positively responded to the AQI in Beijing. Subfigure S1(iii.a) demonstrated that the Baidu index had a negative impact on the local AQI value. Once again, **Hypotheses 1**,**2**, and **3** were all supported.

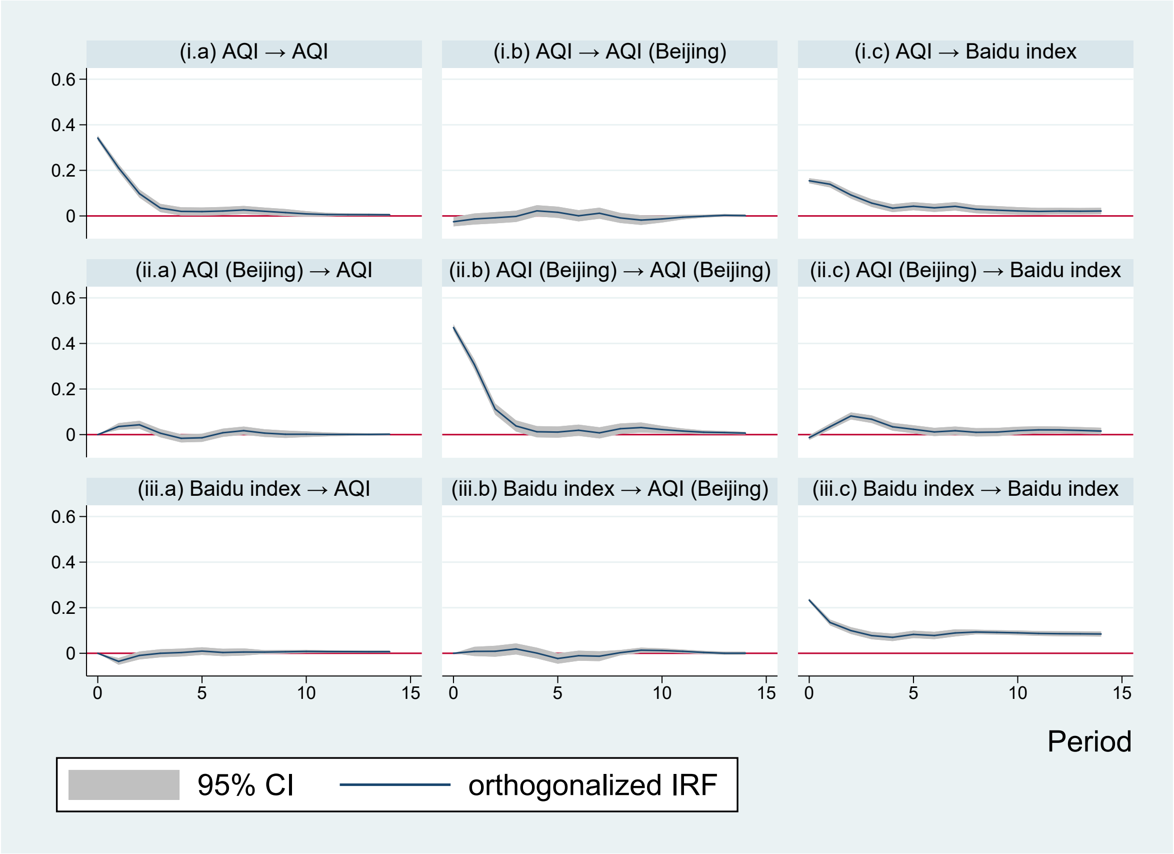

Figure S1: Impulse response figures (IRFs) for the VAR model with Baidu index of keyword "Shanghai $PM_{2.5}$". **Note**: Each subfigure with the title of "X→Y" demonstrates the response of variable Y to an orthogonalized positive shock of variable X. In other words, X is impulse variable, and Y is response variable. One period in the figure denotes one day.
